# The ‘Predelinquent’ and the Community: Psychiatric Surveillance and Predictive Policing in Interwar Berkeley

**DOI:** 10.1093/shm/hkae057

**Published:** 2024-09-30

**Authors:** John Shepherd

**Affiliations:** Department of Philosophy, University of Durham, Durham, UK

**Keywords:** delinquency, psychiatry, surveillance, policing, prediction

## Abstract

Through the 1920s and 1930s, the Berkeley Police Department, renowned as a centre of scientific training and investigation, developed new programmes of predictive policing targeting ‘predelinquent’ youth. Led by Chief August Vollmer, schools, charities, social services and families throughout Berkeley were coordinated in the ongoing detection of early signs of developing psychoses and personality disorders believed to lead to future criminality. Implying a malleable trajectory of habit formation which might be perverted or corrected, predelinquency warranted psychiatric surveillance across the community to assist Berkeley’s police in identifying, mapping and correcting at-risk children. This paper examines how, through the psychiatric category of predelinquency, law enforcement enrolled the community in networks of pre-emptive surveillance with new responsibilities for reporting and correction. In turn, I examine how predelinquency shifted to accommodate various local priorities and anxieties, whereby predictive policing’s conceptions of potential threat or improvability reproduced the boundaries of the normative American community.

## Introduction

In 1923, August Vollmer, Chief of the Berkeley Police Department (BPD), directed police officers, psychiatrists and the public towards a new target, the juvenile ‘predelinquent’: ‘Among the children in our schools today are to be found the gangsters, thugs and murderers of tomorrow, and, inasmuch as we have had pointed out to us by scientific studies and our own observations that the majority of professional crooks were troublesome children long before they became criminals, it behooves the policeman to concentrate his attention upon the problem child during the predelinquent period’.^1^ In Vollmer’s Berkeley, this new imperative to identify and manage the potentially improvable, potentially criminal predelinquent implicated a whole community in both therapeutic care and pre-emptive surveillance, assessment and mapping of at-risk youth. From 1924, Berkeley’s families, public schools, charities, social services, health department and ‘character-building organisations’ provided cases and information for the BPD’s Crime Prevention Division and the city’s Coordinating Council. However, in the hands of these practitioners and informants, ‘predelinquency’ also began to change. The effort to psychiatrically identify future criminality facilitated cooperation across police and community agencies but also readily shifted to incorporate various anxieties and priorities of home, school and neighbourhood. Moreover, in policing this expansive class of predelinquents Vollmer and those he mobilised were also policing the boundaries of a normative community, determining the conditions for inclusion or exclusion.

The surveillance of health and the surveillance of crime have both implicated ‘the community’ as a source of predictive insight, calculation and risk assessment. For David Armstrong, this trajectory in twentieth-century medical history signalled the rise of ‘surveillance medicine’, requiring ‘the dissolution of the distinct clinical categories of healthy and ill as it attempts to bring everyone within its network of visibility’.^2^ Highlighting the problematisation of distinct normal or pathological states, Armstrong thus charted a shift from the diseased body to the disease-potential of the four-dimensional ‘time-community’, characterised by ‘permeable lines that separate a precarious normality from a threat of illness’.^3^ In surveillance studies, Richard Ericson and Kevin Haggerty have similarly pointed to an ascendant model of ‘risk communications’ in modern policing. Responding to public and private fears of insecurity, police have, according to Ericson and Haggerty, increasingly become ‘knowledge workers who join other major social institutions in believing that the world can be made more secure by ever more perfect knowledge of risk’.^4^ Through the lens of predelinquency, this paper will examine the historical intersection of medical surveillance and police surveillance in their concern for ‘precariously normal’ or ‘risky’ children. On one hand, this illuminates how the police have expanded their remit for managing youth, mental disorder and, increasingly, future criminal activity. On the other hand, the case of Berkeley points to the crucial role of the community in shaping fear and suspicion. Berkeley, as a community, was not only scrutinised for signs of incipient delinquency or disturbance but also formed a network of reporting on which police and psychiatrists depended. Further, idealised local and national communities were frequently invoked as a model for determining not only who was at risk but also who was ‘improvable’ and who, conversely, was incorrigibly ‘immoral’ or ‘unfit’.

American contemporaries seeking to understand interwar juvenile delinquency could turn to a range of perspectives implying both optimism, for the correction of behaviour, and pessimism, for seemingly inevitable offending and recidivism. Historians of criminology are familiar with the fatalist biological classification of ‘born criminals’, in Lombrosian criminal anthropology, and institutional segregation of ‘defective delinquents’, by eugenicists who ‘encoded offenders’ bodies with new signs of evil’.^5^ Conversely, Interwar juvenile justice was also characterised by broad therapeutic efforts in movements for ‘mental hygiene’ and child guidance through which ‘maladjusted’ youth might be ‘readjusted’ for productive adult life.^6^ Meanwhile, predelinquency maintained an ambivalent position between optimism and pessimism which, in turn, seemed to necessitate increased oversight of children who might improve or deteriorate.

However, consideration of a child’s future might still be modified by varying judgments of worth informed by class, race or disability. Elsewhere, Margo Horn has described child guidance clinics’ search for ‘more amenable target populations’ and their consequent shift from disadvantaged delinquents to an increasingly private, middle-class clientele.^7^ Miroslava Chávez-García, similarly points to historic discrimination in California’s juvenile justice system where therapeutic programmes to rehabilitate ‘normal’ delinquents disproportionately left vulnerable youths of colour to be pathologised and incarcerated.^8^ More recently, Theo Di Castri has examined later twentieth-century problem behaviour theory in terms of settler-colonial ‘dynamics of assimilation and removal’, wherein treatment is based on links to ‘conventional society’ while those unable or unwilling to assimilate are incarcerated.^9^ Drawing on these perspectives, we must not only trace the extension of potential criminality across predelinquent youth but also consider who was excluded from potential correction and, implicitly, from the future community.

In calling for the psychiatric pre-emption of delinquency, Vollmer drew upon the above criminological and mental hygiene discourses, however, as a police chief integrating prognosis and law enforcement, he was quite unusual. Surveying the field in 1933, psychiatrist and child guidance pioneer William Healy claimed that the BPD was one of only two police departments to seriously engage in such preventative work.^10^ Indeed, this also reflects Vollmer’s contemporary reputation for police reform, being elected president of the International Association of Chiefs of Police in 1921 and thereafter acting as a consultant or temporary Chief for departments in Los Angeles, Chicago, Detroit and other major cities.^11^ In turn, his legacy has been variously lauded and scrutinised by historians. Willard Oliver’s biography of ‘the chief’ thus calls Vollmer the ‘Father of American Policing’, citing his combination of technical innovation (including the introduction of motorised patrols, radio communications, police education and scientific crime scene investigation) and personal virtues (displayed in emphases on non-violence alongside opposition to corruption and racial discrimination within the police).^12^ Hagiographic portrayals are further invoked as a corrective for modern law enforcement, as in Lawrence Sherman’s description of Vollmer as ‘the global example police need to solve the post-Fergusson crisis of police legitimacy’.^13^ Others, meanwhile, have interrogated Vollmer’s progressive legacy, pointing to his disquieting support for eugenics and his early role in police militarisation.^14^ Predelinquency was informed by an eclectic, ambiguous mixture of progressive child-saving, eugenic thought and other impulses to rationalise police power, however, focusing solely on Vollmer does not reflect the policing of predelinquents by a wider community of observers and practitioners. This paper will instead trace how, in practice, the psychiatric notion of predelinquency circulated and shifted between various actors and targets.

In its extension of ongoing behavioural assessment, forecasting and correction, community crime prevention, as set out by Vollmer, resembled the pervasive ‘discipline’ described by Michel Foucault in its ‘uninterupted play of calculated gazes’.^15^ However, this ‘uninterrupted’ discipline does not address the question of how various individuals and agencies were coordinated, or the friction which could occur between them. To explore these dynamics, I instead draw upon perspectives from surveillance studies and science and technology studies (STS) in their description of messier, more pluralistic ‘assemblages’ and ‘actor networks’ which constitute and sustain surveillance and scientific knowledge.^16^ In particular, I use Bruno Latour’s notion of ‘translation’, tracing the shifting knowledge of predelinquency across the community along with its ‘displacements through other actors whose mediation is indispensable for any action to occur’.^17^ Conversely, following the work of Geoffrey Bowker and Susan Leigh Star, the category of ‘predelinquency’ can be considered in its role as a ‘boundary object’ ‘[inhabiting] several communities of practice’ to coordinate crime prevention.^18^ I will thus begin by elaborating on the concept of Predelinquency formulated by Vollmer in collaboration with psychiatrist Jau Don Ball, along with its varying implications for youth, correction and the community. Next, I will turn to the coordination of this community, the actors who made up the preventative network, and their attempts to enforce social, moral or eugenic boundaries. The subsequent sections then follow practitioners in the school and the BPD’s Crime Prevention Division, who negotiated the widening risks posed by disruptive students, ‘shut-in’ children, and the unforeseen female predelinquent. Finally, I will conclude by reflecting on the implications of predelinquency for modern predictive policing and the ongoing demarcation of communities, to be protected or policed.

## Identifying the Predelinquent

Predelinquency, as conceived by Vollmer, initially emerged from the meeting of professionalising currents in both law enforcement and psychiatry. Vollmer, as Berkeley’s marshal from 1905 and then police chief from 1909 to 1932, was preoccupied with the creation of a rationally administered, scientifically trained and technically proficient police force, incorporating ‘knowledge of the fundamental principles underlying human actions’.^19^ Although lacking in formal schooling, Vollmer was, in the words of Julia Liss and Steven Schlossman, a ‘consumate autodidact’, regularly borrowing criminological literature from the University of California, Berkeley.^20^ Correspondence with the city’s probation officer further indicates that Vollmer characterised certain offenders as ‘nervous’ or ‘mentally ill’ as early as 1910 with other anecdotes pointing to his use of Hans Gross’ criminal psychology to infer criminal motivations.^21^ Organising informal lectures for his officers and, later, a three-year police school curriculum on science, medicine and police methods, Vollmer came into contact with long-time psychiatric collaborator, Jau Don Ball. Practicing in nearby Oakland, Ball fostered this medicalised understanding of crime, claiming in later lecture materials that ‘we can unhesitatingly state that at least 60% of our criminals are medical problems’.^22^ Meanwhile, in the *California Law Review,* he presented legal professionals with eclectic examples of unconscious or instinctual criminal motivations, hereditary ‘defect’ and psychosis in the courts, arguing for the necessity of specialist panels in place of judges to evaluate abnormal offenders.^23^ Together, Ball and Vollmer advocated for psychiatric specialists within criminal justice and scientific policing, establishing a BPD ‘psychopathic clinic’ by 1920 and (unsuccessfully) lobbying the California legislature for state and prison clinics to ‘retain in the institution those who are dangerous to the community for as long a period as possible’.^24^

In 1919, Vollmer and Ball planned for the extension of psychiatric prevention into schools. That year, they began investigating ‘problem children’ identified at Berkeley’s Hawthorne Public School from which Vollmer would elaborate his subsequent concept of ‘predelinquency’. The origins of this study are unclear but in March that year Vollmer informed Ball that the school superintendent was ‘ready to put you to work’. At some point the superintendent hoped ‘to establish [a] complete medico-psychological clinic in connection with the school department, and have every child in the schools examined’ but, for the present, their attention would be ‘confined to the training of school teachers and the examination of the notoriously unfit’.^25^ Though detailed records of this Hawthorne study are lacking, Vollmer wrote to others of Ball’s recent school survey involving ‘psychological psychiatrical and general medical examinations’ as well as a social survey made by the local Red Cross Society under the direction of the Berkeley Dispensary. Meanwhile, police records were scoured for any information from the ‘family, developmental and industrial view-points’ to be assembled in a single file.^26^ As will be discussed shortly, initial investigations at Hawthorne thus pointed to the wider networks of cooperation upon which subsequent pre-emptive programmes depended.

Reporting on the Hawthorne study in 1923, Vollmer published a brief article simply titled ‘Predelinquency’, one which, nevertheless carried wide-ranging implications for children’s uncertain futures and the preventative responsibilities of police and community. Beginning with an overview of prior criminological authorities, he argued that ‘the chief factor in juvenile delinquency is the unstable or stunted mental make-up of the children’. This was, Vollmer claimed, now supported by Ball’s successful detection of such mental characteristics in ‘predelinquent’ children at Hawthorne. The school survey supposedly confirmed the expectations of both police and criminologists: that ‘the habitual offender’s criminalistic tendencies were displayed by non-conformity to regulations at a very early age’.^27^ Some illustrative cases were briefly described. Case I was a habitual truant, prone to violent fits of anger and unable to accept reprimand, ‘a stupid boy’; Case II was 'very bright' but emotionally unstable, crying easily and continuously fidgeting as well as lying regularly; finally Case III, according to his mother, was different from other children ‘being seclusive and solitary, a shut-in individual’, showing dislike for others of his age and successively breaking his own toys before moving onto injuring animals and, finally, throwing rocks at younger girls, resulting in his dismissal from school.^28^

Such illustrative cases would have been familiar to contemporary child guidance practitioners, however, Vollmer went further in outlining a combined police, school and community programme of pre-emptive surveillance. Schools would provide the earliest data warning of potential behavioural issues which could then be recorded on maps displaying the residences of ‘problem children’. Colour-coded pins would categorise these children in terms of different behavioural and psychological risks such as blue for ‘troublesome’, red for ‘immoral’, black for ‘habitual truant’ and white for ‘feebleminded’.^29^ As well as identifying targets for therapeutic intervention and monitoring these maps would also inform police patrols, acquainting beat officers with local problem cases and their potential for delinquent or other disruptive activity. Finally, Vollmer pointed to the imperative for reporting and correction beyond law enforcement, using predelinquency to enrol and coordinate the entire community:

Thus, armed with facts, not fancies, and with a constructive programme for the mental, physical and moral health of the subject, the policeman is in an enviable position insofar as the future of the child is concerned. With a higher social ideal, with a broader vision of his position in the community and with the power of the state behind him, he can command assistance from parents, teachers, preachers, recreation supervisors, welfare agencies, dispensaries, and every other health and character-building organization in the community.^30^

Before turning to the role of this community network, Vollmer’s call to action must be placed in the context of contemporary anxieties for youth and their development. As noted in the introduction, criminal conduct and other emotional or behavioural problems increasingly prompted attempts at therapy and prevention in interwar child guidance clinics, spreading to 42 American cities by 1933 with further international reach from the later 1920s.^31^ Kathleen Jones’ study of the first such clinic, Boston’s Judge Baker Foundation established in 1917, also notes the widening scope of clinical intervention, extending from court cases to the management of ‘normal’ emotional, educational and vocational concerns wherein ‘the troublesome child acquired an “everyday face”’.^32^ Meanwhile, Margo Horn’s history of the child guidance movement and its promotion by the Commonwealth Fund connects this extension to ‘the imperatives of professional groups whose roles and services were in part justified by this broad view of the at-risk child’.^33^ Predelinquency thus reflected the expanding remit of contemporary psychiatry for the prognosis and prevention of perceived moral and social ‘failure’, understood in terms of ‘maladjustment’. Introducing the first issue of *Mental Hygiene*, William A. White described the ‘passing of insanity as a medical concept’ and psychiatrists’ consequent freedom to move beyond the asylum and assist in the ongoing ‘adjustment’ of individuals to their surroundings.^34^ Indeed, the career of Jau Don Ball in Berkeley reflected this interwar professional shift, encompassing ‘prophylactic criminology’ but also the treatment of convalescent soldiers, after which he also offered his services to California businesses in screening employees for the ‘agitator type’.^35^

The threat of incipient mental, and consequently social or criminal, disorder not only seemed to justify wider oversight, but also carried ambiguous implications for which children were deemed ‘normal’ or ‘abnormal’, improvable or unimprovable. In ‘Predelinquency’, Vollmer indicated an eclectic mixture of nature and nurture with varying potential for correction. His above correspondence with Ball had explicitly prioritised the identification of the ‘unfit’ while the article subtly implied the possibility of eugenic segregation for some: ‘we cannot expect to undo the follies of past generations in a few years of careful training, nor perform such miracles as furnish intelligence to an imbecile, but we can help every child by securing for him the environment best adapted for the development of his potentialities’.^36^ Conversely, predelinquency pointed to the malleability of childhood and consequent promise of ‘character building’ in these formative years which might still avert a criminal future. In contrast to adult rehabilitation, which Vollmer elsewhere described as ‘hopeless, and in the main, fruitless work’, juvenile predelinquents were distinguished from mature recidivists precisely because their futures remained unresolved.^37^ This implied that pre-emptive action was potentially effective and, hence, absolutely necessary.

Subsequently, Vollmer continued to advocate child guidance and habit training alongside eugenic instruction against ‘bringing defective children into this world’.^38^ As will be seen, community crime prevention in Berkeley similarly differentiated between risky youth, who required sympathetic treatment, and those deemed ‘unfit’. This variously eugenic management of predelinquency was in keeping with contemporary ‘progressive’ interests in the ‘rational’ control of reproduction, as well as California’s own record of involuntary sterilisations (totalling an estimated 20,000 between 1909 and 1979, the highest number of any US state).^39^ It was also in keeping with the eclecticism of child guidance in its focus on the varying needs of ‘the individual delinquent’.^40^ Indeed, though the creation of maps to track predelinquency is reminiscent of Chicago sociologists’ and social psychiatrists’ subsequent mapping of crime and mental illness, prevention in Berkeley remained focused on individuals and their potentialities.^41^ As in the specificities of the three example cases given by Vollmer, predelinquency might be ascribed not only to children who were too disruptive and ‘ungovernable’ but also those who were too shy or otherwise concerning to practitioners on various grounds. In part, this reflected psychiatry’s ongoing ambivalence regarding the extent of ‘natural’ rebellion and misbehaviour to be expected from young boys and adolescents especially.^42^ It also points to the plurality of claims upon children and their futures from across the community.

The school study suggested that pre-emptive surveillance and prevention could not depend on police and psychiatrists alone. Berkeley police historian Alfred Parker recalled Hawthorne as a successful ‘experiment in cooperation’, extending to teachers, social agencies and, apparently, even parents.^43^ Earlier, Vollmer had voiced his concern that Ball’s questionnaire might raise active opposition and stressed the need to avoid a ‘militant’ campaign. In particular, the ‘Public Schools Protective League’ was identified as a threat to the study, having reportedly advocated ‘the prevention of examination of school children by medical authorities’ and resisted such examinations in Los Angeles. Vollmer nevertheless hoped that parents would ‘recognise the benefits that accrue from scientific examinations’ over the claims of ‘Protective League influencers’.^44^ Indeed, Vollmer later wrote to Mrs. Queen Layer, Secretary of the Berkeley Federation of Mothers’ Clubs and Parents Associations, gratified to hear that they had endorsed the plan for a psychopathic clinic attached to Berkeley’s schools.^45^

Parental cooperation may be further illuminated by Vollmer’s irregular correspondence which suggests that he was, by this time, regarded as an approachable authority on child behaviour. Regular appeals for expert evaluation reached Berkeley from across the bay area and wider California. Thus, replying to J.F. Fidler in November 1920 Vollmer advised that his son’s thefts might be due to factors detectable only by careful expert examination.^46^ Two months earlier, he had advised Mrs. M. Brady on treatment for her son, suggesting that Ball was known to ‘waive large fees where he feels the need for his services’.^47^ These services were also recommended to Mrs. L.C. Weymouth in July 1921. Here, Vollmer again stressed that ‘it will be utterly impossible for me to assist in guiding your child without having all of the facts regarding the youngster’s physical and mental makeup, plus his reactions to the different environments that have surrounded him since birth’.^48^ Vollmer’s responses regularly re-iterated that therapeutic assistance was conditional on providing full information to qualified experts. For these parents at least, Vollmer’s position between both police and psychiatric authority promised potential access to experts, decision makers and, thence, leniency for their children. Faced with concerning or criminal behaviour, psychiatric assessment could be a preferable alternative to the prospect of further offending and court involvement. As will be seen, however, not all cases were thought to warrant this kind of guidance and parents could themselves be regarded as problems as well as participants in community crime prevention.

The subsequent extension of police and psychiatric power over the predelinquent was not straightforward. Considering the shared anxieties of parents and other practitioners leads us to what Jones has called ‘the problem of authority’ in child guidance.^49^ The following sections will chart the enrolment of the various actors in pre-emptive surveillance, assessment and intervention, moving beyond the presumed authority of police to define civil disorder and, conversely, the presumed authority of psychiatrists to define mental and behavioural disorder. By turning back to the priorities of the community Vollmer so frequently invoked we can further explore the role of predelinquency in demarcating its boundaries.

## Coordinating the Community

From 1924 plans for cross-community action against predelinquency were put into practice through the newly formed Berkeley Coordinating Council, intended as a point of contact between health, education, law enforcement and other social agencies. That year, Vollmer had begun meeting regularly with the chief of Berkeley’s health department, Dr William P. Shepard and assistant superintendent of Schools Virgil E. Dickson, eventually formalised in a weekly one-hour conference to discuss shared problem cases. In 1925, they would be joined by Elizabeth Lossing (née Anderson), a Berkeley local returning from the New York School of Social Work to direct the BPD’s newly established Crime Prevention Division (CPD) targeting predelinquency. Moving beyond Vollmer’s attitudes discussed in the previous section, the members of the coordinating council brought with them subtly different priorities in managing the predelinquent. Dickson’s school programme approached broadly construed problem youth with an inclusive goal of training for citizenship, extending to the education of parents in their child-rearing responsibilities. Conversely, though sources for his involvement and attitudes are lacking, Shepard seems to have favoured institutional, possibly eugenic measures. Meanwhile, Lossing’s social work training was reflected in her wider engagement with community agencies and families, eventually encompassing the wider concerns of mothers and daughters in Berkeley. Her instruction in New York by prominent behaviourist psychologist John B. Watson may also account for the Berkeley programme’s increased emphasis on reporting and correcting behaviour, rather than the psychoanalytic models increasingly adopted by child guidance clinics elsewhere.^50^

Along with these key figures, meetings of the Coordinating Council would, by 1932, include the superintendent of social service, visiting teacher, executive secretary of the welfare society, and the director of playgrounds.^51^ However, the coordination of these overlapping spheres also raised the prospect of disagreement and contested responsibilities. Dickson, later writing as chair of the Council thus stressed its necessarily ad hoc, informal organisation:

We are wise enough not to try to dictate. If our coordinating council were made a requirement by the city charter and we were forced to vote on interdepartmental policies, we would break up in a row, and would need the rest of the police department, in addition to the chief, to settle our differences.

Elsewhere he elaborated that, ‘no one in the council has power to give or take except that which he is willing, yet in that group rests the authority to say, "I will send a police officer this afternoon to the house" or "the child will be given a careful observation at the health centre" or "he can be placed in a special class tomorrow"’.^52^ The eschewal of formal proceedings was not only intended to satisfy separate departments but, further, reflected Vollmer’s own preference for a pluralistic, participatory programme of therapeutics. Discussing the plan in November 1925, psychiatrist Herman Adler praised Vollmer for avoiding the error of an ‘autocratic and paternalistic undertaking’, instead seeing in Berkeley ‘a good democratic and progressive program’.^53^ Responding, Vollmer similarly dismissed paternalism in favour of public education and participation, hoping that ‘the plea for help will come from the people and we shall be in a position to provide the necessary assistance’.^54^ Indeed, this was in keeping with his earlier ‘positive police plan’, described by Liss and Schlossman, which aimed to draw upon the community’s knowledge of incipient threats not only by acquainting officers with locals and businesses on patrol but also by encouraging the proactive questioning and reporting of suspicious persons and strangers to Berkeley.^55^

Thus, coordination against predelinquency was articulated in terms of non-coercive assistance for a willing public, however, ‘democratic’ language was also mirrored by exclusionary drives to identify and remove external and internal threats. The reference to ‘democratic’ crime prevention, in turn, leads to a further crucial point: through their participation in the Coordinating Council, Vollmer and other officials conceptualised Berkeley as an ideal, morally cohesive, community, along with their responsibility for preserving its integrity. Berkeley could apparently take on the shared task of therapeutic community service in contrast to larger metropolises, viewed as sites of ethnic, cultural and moral deterioration. In his letter to Vollmer, Adler continued that ‘the fact that it is Berkeley, and not Chicago or New York or even San Francisco, greatly adds to the probability of success’, citing the difficulties of ‘a larger and less homogenous community’.^56^ Vollmer optimistically reassured Adler that success in larger cities depended simply on willpower and leadership but elsewhere contrasted Berkeley with apparently unsettled urban centres. Years earlier he had attributed Berkeley’s lack of ‘dance halls, deseased [sic] prostitutes, street walkers and the like which are common in every other city’ to the attraction of ‘more thickly congested sections’ in nearby Oakland and San Francisco.^57^ Later correspondence similarly pointed to Chicago, Detroit, Los Angeles and New York, citing ‘social unrest’, ‘social indigestion’ and ‘the large constant influx of mixed nationalities’, in contrast to ‘other more stable communities’.^58^ Re-adjustment of youth to laws and shared social expectations within the ‘stable community’ of Berkeley was thus carried out in light of the often-unstated, parallel anxiety of perceived urban vice, cultural disunity and foreign encroachment.

Work to intercept and correct predelinquency in Berkeley thus aimed to defend and reproduce the values and behaviours of a predominantly white, middle-class population. In her 1932 report to the US Department of the Interior on Berkely’s programme of behavioural adjustment, Elise H. Martens described the university town as ‘a centre of cultural opportunity and professional service’.^59^ Meanwhile, opening her report on the CPD with statistics from the 1930 census, Lossing wrote that ‘if figures help, picture Berkeley’s population as being 94.5 per cent white, 2.6 per cent negro, 2.9 per cent other races’.^60^ In fact, Vollmer had already mobilised his police department to target racialised threats. As Julian Go notes, Vollmer’s initial election as marshal in 1905 rested in large part on his prior military service in the Philippine-American War and on local fears of increasing Chinese immigration. Subsequently, Vollmer equated Chinese ‘crooks’ with Filippino insurgents through his early police campaigns targeting Chinese laundries and other potential ‘vice dens’.^61^ Distinctions between the protected community and external threats continued in the subsequent work of Lossing’s CPD. A description of its remit, written in 1927 for the International Association of Chiefs of Police thus included ‘causing to be deported undesirable aliens’ and ‘arranging for commitments of defective, dependent or delinquent persons to public institutions’.

Opposite those who were to be excluded, the same letter outlined far more extensive responsibilities of the CPD for those to be included in community correction, as well as for internal threats to their mental and moral development. known offenders were joined by potential delinquents grouped under several categories: ‘problem children’ identified in schools, neighbourhoods and welfare agencies; ‘handicapped children’ identified by physical, mental or moral ‘defect’ as well as dependency; children of ‘degenerate or criminal parents’; those living in ‘unwholesome environments’ and finally, discharged psychiatric patients. Law enforcement thus looked for criminal propensities in a range of background traits or ‘defects’ while also assuming responsibility for supposedly criminogenic spaces.

The mapping of at-risk youth further encompassed homes, locations and neighbourhood situations considered deleterious. ‘Defective home conditions’ to be monitored or corrected included those reported for ‘immorality’, neglect, dependency, poverty, ‘excessive quarrelling’, alcoholism and ‘insane, feebleminded or degenerate persons’. Meanwhile ‘defective neighbourhood conditions’ ranged from gangs and sites of gambling, bootlegging or prostitution to ‘anarchist societies’, ‘racial quarrels’ and ‘demoralising individuals in community’. In turn, public spaces of juvenile recreation such as dance halls, playgrounds and motion picture houses were marked out for supervision. Opposite the surveillance of these threats to community cohesion, crime prevention responsibilities also described the active promotion of community centres, ‘character building organisations’, ‘civic betterment organisations’ and educational campaigns targeting juveniles and parents. However, community crime prevention remained backed by police assistance to health, school and welfare departments, providing follow-up officers to ‘obtain support’ from families, prosecute cases and ‘bring to bear upon problem children the constructive and rehabilitating forces of health, educational and character building agencies’.^62^

Emphases on the coordination or participation of the community in mutual assistance were thus qualified by police power offered by the BPD and its Crime Prevention Division. In some illustrative cases of the Coordinating Council in action, Martens’ report thus discussed the extension of educational, health and welfare services to a ‘serious problem child’ whose parents were thought to need instruction:

Any one of the workers going into the home may need the moral support of the law. A policeman in uniform merely goes along. The uniform does the work without the necessity of words.

These cases also point to those situations considered threatening enough to warrant more forceful intervention. The welfare society presented one such family ‘getting rapidly worse’ with over 20 people in one home, 13 of them juveniles, with ‘filth and living… conditions so horrible that the younger children have no choice for developing into anything but delinquency and crime’. Combining reports of illness, school disciplinary records, police files and the recreation department’s reports of ‘trouble on the playground’, the Coordinating Council successfully persuaded the juvenile court judge to break up the home and place the youngest children in foster care. As well as environmental ‘defect’ the Coordinating Council’s concerns also extended to the removal of mental ‘defect’, mirroring Vollmer’s own variously eugenic views. The schools thus called attention to a ‘feeble-minded’ family, warning that ‘children were limited only by the calendar and biology’. A ‘complete history’, gathered by members of the council, was subsequently presented to a judge and the mother committed for sterilisation. Employing typical eugenic rhetoric, this case concluded that ‘society has been saved the burden of additional dependents from that source’.^63^

As the foregoing discussion illustrates, Berkeley’s ‘good democratic and progressive programme’ did not preclude the exercise of state power over those thought ‘unfit’ to participate in that programme. Positive measures to correct and care for youth had their counterpoint in negative measures to contain or cut off the ‘pathological’ or ‘defective’. Indeed, the same report detailing the above sterilisation soon went on to describe examples of sympathetic guidance in schools. Here, it was instead hoped that a willingness ‘to probe deeply, gently, patiently, understandingly’ might reveal the child’s struggle and ‘bring back into the picture the harmony and beauty that belong there’.^64^ This contrast can be understood in light of Vollmer and the Coordinating Council’s repeated reference to their responsibilities for the community. Stability and cohesion, sometimes through forceful exclusion, were thought necessary to preserve the therapeutic potential of this community. Meanwhile, turning to these corrective efforts in the school and the CPD in more detail reveals how expansive categories of predelinquency and problem childhood could be variously understood by different practitioners.

## From School to Home

Virgil E. Dickson, as director of Berkeley Public Schools’ Bureau of Research and Guidance, not only looked to the correction of predelinquents but, further, to the creation of citizens. Broadly construed problem youth were thus to be guided for future participation in both the local and the national community. Since 1918, Dickson had already introduced systems of classification and ability grouping as well as vocational guidance, and readily offered the school as a source of information on predelinquent behaviour.^65^ Looking to correction, principals and teachers were, from the fall term of 1928, asked to report all serious behaviour problems from kindergarten to the ninth grade as part of a study in the adjustment of problem behaviour. The resulting 365 problem children were then divided into an ‘experimental group’ of 120 to receive focussed assistance while the remainder formed a control for later comparison.^66^ A better understanding of 'undesirable behavior symptoms' and their early correction was necessary to avert ‘the tragedy of the unadjusted school child’ and ‘the even greater tragedy of the psychotic adult and the social delinquent’.^67^ Conversely, the prevention of potential delinquency became part of school counselling’s broader social responsibility to all children’s futures and ‘the purpose of the school to train every child to be a cooperative citizen’.^68^

In this behavioural study, Dickson depended on reports from teachers but also evidently attempted to regulate their potential for error or deviation from expert perspectives. Other information was collated on the child’s social, medical and developmental history, family, recreational practices and neighbourhood. However, serious problem behaviour had to first be identified in the classroom, defined as any ‘which varies sufficiently from normal behaviour to cause the teacher to feel that the child cannot be managed satisfactorily with the group’. Through termly behavioural and personality evaluations teachers thus recorded ‘retardation’, truancy, ‘sex difficulty’, stealing, fighting, lying, ‘nervous instability’, cruelty, reticence or ‘any other behaviour deviating from normal’.^69^

The enrolment of teachers in periodic behavioural assessment reflected Dickson’s stated conviction that ‘every teacher should be, to a certain extent, a counsellor’.^70^ However, subsequent forms for periodic reporting attempted to confine them to specific behavioural problems which were now uniformly codified and assigned a numerical score based on mean ratings of ‘seriousness’ by mental hygienists and educational psychologists.^71^ ‘Writing notes’ was thus given an assigned value of 2 while more serious incidents like lying, truancy and stealing were, respectively, scored 12, 11 and 14. Amongst sexual concerns, ‘vulgar speech’ (scoring 9) was rated slightly more serious than masturbation (8) but scored less than sexual pictures or stories (10) and reported heterosexual activity (13). Some of the most serious problem behaviours, according to expert ratings seemed to reflect emotional instability, including ‘temper outbreaks’ (scoring 13) and ‘weeping’ (scoring 14). Any of these ratings could be multiplied depending on whether the behaviour was isolated, occasional or frequent.^72^ The result, according to Elise Martens in her evaluation of Berkeley, was that ‘it became a simple matter of mathematical calculation to compare the groups from term to term and to note the progress made from the first to the last semester reported’.^73^

As in Porter’s analysis of numerical objectivity in science and policymaking, ‘trust in numbers’ conversely reflected distrust in teachers’ abilities to accurately assess ongoing problem behaviour in their classrooms.^74^ Even so, Dickson still worried about experts’ and teachers’ divergent priorities in reporting behavioural risks. Speaking in June 1931, he compared the opinions of 24 psychologists working in child adjustment to Berkeley’s teachers, noting that ‘inattention’ or ‘disorderliness’ made up the vast majority of difficulties reported by the latter. While teachers ranked these behaviours alongside moral offences such as lying or stealing as especially serious, Dickson found that amongst psychologists these types of misbehaviour were not prioritised. Rather, signs of nervousness, sensitivity and depression were considered the most serious indicators of future maladjustment, behavioural difficulty and psychosis. Teachers, prioritising the order and routine of the classroom arrived at different conceptions of problem behaviour, diverging from expert fears of the dangerous ‘shut in’:

if the psychologists and mental hygienists are right those things which cause the most disciplinary troubles to the average teacher are natural to a normal child. As a matter of fact they are evidence of a personality that is endeavouring to express itself and grow strong, while those traits that cause the least trouble to the average teacher are symptoms of a personality that is on the road to trouble.^75^

Returning to the experimental group of problem cases in 1931, Dickson saw further confirmation of this divergence. The most frequently reported problems were lack of application, emotional difficulty, defiance of authority and offence against society however Dickson claimed that, in a sample of non-problem children, 50 per cent displayed ‘reticence or timidity’, unreported by teachers.

Along with uneven behavioural reporting, Dickson noted a lack of unifying explanatory factors in the problem group. It included 84 boys and 25 girls encompassing all grades and ages with an IQ range of 50–160 while differences in SAT achievement were considered insignificant. Summarising, Dickson quickly noted: ‘Problem cases predominantly boys. All ages. All I.Q. Achievement normal. No Reliable Physical difference’.^76^ Perhaps his only firm conclusion was an emphasis ‘in environmental as against hereditary factors’.^77^ This was reflected in the illustrative cases Dickson chose to describe the connection between abnormal behavioural development and parenting or home conditions. One girl of nine apparently became effectively mute after a ‘serious emotional disturbance in early childhood’ in ‘a clear case of problem parents’. Another boy of 12 lied and stole with ‘no basis of need’, owing to a broken home, while other cases showed poor health or malnutrition.^78^ One particularly extreme case, a ‘brilliant’ 12-year-old boy, was allegedly ‘whipped so severely by the father that he has become almost calloused to the pain’. Here it was concluded that ‘after more than a year of work the boy is still a serious problem, irreparable harm has probably been done to the boy’s personality’.^79^

Dickson soon sought to intervene directly in the home situations behind his problem cases through radio talks, intended to educate parents on the risks of child development. From 1932 to 1936, he offered ‘everyday’ anecdotes from his experience of school guidance counselling in ‘Mind Ways: Stories of Human Behaviour’. His first broadcast thus re-iterated that ‘these problems are yours and mine as parents’.^80^ Subsequent episodes described ‘the dangerous child who causes no trouble’, ‘the runaway girl’, ‘the boy who never grew up’ and myriad other cases for the education of anxious parents. Often these concluded by reiterating the natural antagonism of childhood and consequent necessity of wholesome, open and psychiatrically informed engagement with the emotional needs of youth, mirroring contemporary child guidance’s literature in its emphasis on ‘a companionable or democratic rather than authoritarian relationship with a parent’.^81^ The anxious prevention of delinquency and problem behaviour now turned back to homes and families in their wider responsibilities for training, informed by scientific understanding of the child.

Other broadcasts reflected both Dickson’s interest in citizenship training as well as his listeners’ responsibility for the potential criminal. In February 1934, he thus told the story of ‘John’ who used to be ‘the worst devil I ever knew’ but who later ‘became a loyal, patriotic citizen and is now a respected man in his community, state and nation’.^82^ In September 1936, another notable broadcast asked ‘Is One Born to be a Criminal?’ and described ‘Bob’, a 15-year-old police problem whose father blamed maternal inheritance for his actions. However, Dickson’s response, in contrast to earlier discourses on the ‘defective’ delinquent, pointed to parental responsibility: ‘I do not believe that there is any scientific evidence to warrant the belief that a child inherits his criminal tendencies. I believe that your son, Bob, developed his habits through the way he was managed rather than from the stock from which he came’.^83^ His words here, and the parent education broadcasts they appeared in, both pointed to expanded notions of preventative and corrective responsibility extending from the school back to the home in their maintenance of moral community.

## The Crime Prevention Division

While the school facilitated early detection and ongoing reporting of possible behavioural problems, from 1925, the BPD’s Crime Prevention Division undertook proactive police work, negotiating a range of claims upon those considered predelinquent. The previous year, Vollmer had approached Elizabeth Lossing with a new role leading the CPD and ‘dealing with the pre-delinquency problem’. Beyond the police department, however, this would be ‘an effort to concentrate these forces that deal with the health, education and morals of the children upon the problem child long before he reaches the police station’.^84^ As she prepared to leave for Berkeley, Vollmer wrote to her on the pooling of police, school, health and welfare records under the new coordinating council ‘with the hope that the massed facts thus obtained may suggest remedial measures’. Maps were being prepared to display this accumulated data on Berkeley’s problem children to allow an intelligent, effective and coordinated response. Vollmer’s ‘plan of attack’ thus approached the city of Berkeley and its community as a field of both risk and information gathering, with aggregated reports directing officers and other agencies to particular individuals or locations.^85^

Reflecting Vollmer’s initial outline of ‘Predelinquency’ and the subsequent work of the Coordinating Council, pre-emptive investigation and intervention could range from informal acquaintance and collaboration between local organisations up to direct police and legal intervention. In a report on the CPD in 1936, Lossing herself stressed openness to and reliance upon public participation as well as acknowledging various preventative priorities, ‘organising the city for work with predelinquents and young delinquent children’ by first ‘interpreting the aims and ideals of crime prevention in the community’.^86^ Further, the CPD regularly presented delinquents, parents and other interested parties with the possibility of avoiding formal proceedings. Lossing thus stressed that ‘Our “unofficial” action… is in keeping the situation in our own department, but getting all the community help we can in adjusting the problems of the human being with whom we are working’.^87^

As in the public schools, crime prevention merged with the broader goal of fostering normal development, intervening in the home and neighbourhood through educational materials and ‘character building agencies’ to ‘replace undesirable habits with socially acceptable ones’.^88^ Once again, anecdotes of the ‘typical’ case pointed to parental responsibility for behavioural problems. ‘Ronald’ was noted for his ‘poorer home’ and ‘inadequate parents’ and ‘Harry’s improvement was undone by ‘a father who fails him’ while ‘Dick’ had an absent father and a history of physical abuse by elders. In some cases, Lossing indicated that ‘nothing short of a complete separation of the child from his family will avail us anything’. Conversely, these examples pointed to the potential of improvement and flourishing in ‘an ideal environment’.^89^ This work was, however, formally split between gendered spheres of preventative responsibility. In the CPD, Lossing directed efforts to manage and correct all female cases along with boys up to the age of 12. Meanwhile, the correction of older boys was mediated by the discretion of police officers through their neighbourhood patrols. In keeping with contemporary notions of female suitability for social work with families and juveniles, Vollmer had previously described the role of policewomen as new specialists in ‘that great untouched work, predelinquency’.^90^ However, troublesome male adolescents in the community would remain under the masculine guidance of local beat cops.

The plan to identify and map predelinquents had implied a more complete knowledge of psychiatric risk for patrolling officers, however, the actual extent of police oversight and intervention into the lives of Berkeley’s adolescent boys is difficult to evaluate due to this discretionary nature. As David Wolcott has argued, American police regularly shaped juvenile justice through their informal decisions to target, arrest or release offenders as well as through their claimed first-hand experience in recognising and disciplining juvenile delinquents.^91^ In the case of Berkeley, Lossing pointed to some individual patrol officers who apparently took up Vollmer’s call to engage the community and guide adolescent boys. Sergeant C.H. Ipsen was thus noted for delivering food and clothing to particular families as well as organising the creation of a baseball park on derelict land, providing local boys with other sports equipment. Meanwhile officer A.E. Riedel had led hikes and swims with the YMCA for adolescent cases under his charge and sponsored a football team for troublesome boys. Lossing recounted another instance where he had ‘succeeded in getting a Japanese gang and a colored gang, formerly at sword’s point, organised into live wire football teams’. Other activities promoted by officer Riedel included a reading club for 16- and 17-year-olds as well as organising a series of ‘real dress-up dinner parties’ for young couples.^92^ Joining Dickson and the CPD’s promotion of an ‘ideal’ home environment, these officers, at least, looked to the creation of an ‘ideal’ neighbourhood through wholesome sporting, recreational, social and even romantic activities organised under informal police supervision.

Although the discretionary treatment of adolescent boys by patrol officers remains obscure, Lossing provided more comprehensive information on the CPD’s own activities along with the wide networks of cooperation it relied upon and the new meanings that were consequently attached to predelinquency. Over 10 years of operation, Lossing had been involved in 2,563 juvenile cases, involving approximately 7,500 visits from juvenile and adult ‘probationers’ to the CPD as well as over 7,000 visits to schools, hospitals, social agencies, courts and homes.^93^ This effort accordingly drew upon assistance and information across a network of local volunteers and reports. Lossing reported working with over 400, volunteers drawing on freelancers, church groups, local women’s organisations and the YMCA and YWCA of the University of California.^94^ In turn, school officials, neighbours, social agencies and industrial or commercial concerns were responsible for referring hundreds of juvenile cases to Lossing and her staff while dozens had even referred themselves. Most notably, by 1936, parents had referred 125 male and 489 female juveniles to the CPD and had been the most regular source of referrals until 1933 when procedural changes required BPD officers to copy all reports concerning juveniles to Lossing and her staff.^95^

Responding to the CPD’s promises of unofficial assistance, parents and various other informants sought out pre-emptive police involvement, overwhelmingly for the purposes of regulating female behaviour. In earlier correspondence with one institution for girls, Vollmer had speculated that ‘this department will probably not have more than two or three cases of delinquent girls that would be handled by your institution during the period of a year’.^96^ Lossing herself referred to the impersonal delinquent as ‘he’ with example cases of ‘Tom’, ‘Dick’, ‘Jim’ and so on. Yet, the majority of juveniles referred to the CPD were female, 1,689 as compared to 874 males. Meanwhile, Lossing had unexpectedly found herself dealing with 1,905 adult women including prisoners, probationers and those suffering from mental illness along with ‘women presenting their versions of domestic controversies’ and requesting assistance.^97^

Based on Jau Don Ball’s case notes, the residents of Berkeley had evidently mobilised his earlier BPD ‘psychopathic clinic’ with similar hopes of controlling women thought by the community to be unstable, disruptive or sexually immoral. ‘Mrs. B.’ was thus reported as ‘a cause of much annoyance to her relatives, the community and the police department’, evicted by her son for ‘making herself obnoxious’ and eventually committed to an institution following the BPD’s involvement.^98^ In the case of ‘Mrs. R’, ‘a menace to the happiness and welfare of the community’ the perceived threat was promiscuity and interference with other families.^99^ Here Ball cited complaint letters on her reported attempts to seduce married men in Berkeley along with a local doctor who called her an ‘overdeveloped sexual freak’.^100^ An officer responding to another complaint suggested that she may ‘reach collapse or breaking point and become insane’ before stating: ‘Her ideals of morals are not in keeping with the customs of the country’.^101^ The Coordinating Council’s campaign against predelinquency and purported community problems had similarly extended to the oversight of sexual behaviour and removal of suspected risks to wider physical and moral health. Writing to Vollmer on the ‘the problem in the case of Jean Stewart’ in 1925, health department chief Shepard, working with a local school nurse, discussed allegations of Jean’s ‘promiscuous relations’ which might be expedited by a diagnosis of venereal disease. By obtaining parental consent for a blood Wasserman test for syphilis or smear tests in routine school examinations, Shepard wrote ‘our chances should be very good to be able to exclude her from school and hasten her admission to an institution’.^102^

In the above cases, promiscuity was held to threaten the health, morals and families of the community, in turn warranting removal. Meanwhile, in the CPD Lossing encountered parents and others directly referring young girls deemed morally at-risk, so that they might be brought back under the regulation of community and family. Reasons given for referral are suggestive of the shared anxieties which thus constituted the female predelinquent. While boys formed a majority of cases for stealing and disorderly conduct, girls were more commonly referred for every other listed offence. These included 213 girls reported under ‘truancy, runaways, missing persons’ and 187 for sex offences compared to 77 and 26 boys, respectively. The most common reason listed for female juveniles’ referral was the possibly euphemistic ‘ungovernable—beyond parental control’, encompassing 377 girls compared to 82 boys.^103^ Lossing herself characterised the female delinquents as ‘runaways, some theatre mad, emotionally immature, self-centred, domestically unhappy, poorly endowed, poorly mothered’.^104^ The concern for young girls’ morals, proper parenting and the flouting or flight from expectations of home and community thus presented the CPD with its largely female caseload, along with alternative characterisations of predelinquent youth.

## Conclusions

When Vollmer articulated the threat of predelinquency he did so with the intention of mobilising his community through a shared psychiatric model of pre-emption and correction or control. However, the shared target of predelinquency also proved malleable for various actors in this surveillance network. Alongside the potential for future criminality, psychiatric conceptions of the dangerous, maladjusted ‘shut in’ child were joined by teachers’ priorities to control the most disruptive or defiant schoolchildren. Police supervised a range of young misdemeanants and gang members while parents referred promiscuous or ‘ungovernable’ daughters to Lossing’s crime prevention division. In facilitating cooperation, predelinquency, like any good prophecy, thus remained necessarily vague. Firstly, the ambiguities inherent in forecasting children’s futures suggested the potential for both criminality and corrective intervention, making wide-ranging structures of surveillance and governance imperative. Secondly, predelinquency remained open to diverse priorities and anxieties in the identification of transgressive or abnormal behaviour, offering categories by which to access decision makers in policing, health and education.

However, in the process, Vollmer’s original target, ‘predelinquency’, had also become more diffuse, targeting offenders, broadly construed problem youth, families and ‘defective’ individuals with variously ‘character building’, educational, recreational, social service or eugenic interventions. Although the absence of further sources makes any subsequent evaluation of these institutions difficult, the informal acquaintances and interventions of coordinated agencies and the CPD might be disrupted when their ‘stable community’ lost key resources. When Lossing wrote her report in 1936, she briefly discussed the economic constraints of the depression, which had seen the closure of Berkeley’s child guidance clinic and the reduction of school psychiatric services. Without reports from these sources, the CPD was discontinuing its annual tabulation and analysis of physical, mental and social factors amongst juvenile offenders.^105^ When setting out his ‘plan of attack’ to Lossing in 1925, Vollmer had imagined the mapping of predelinquency further extending to comparative studies evaluating prevention through ‘every agency that can be useful in elevating the mental, moral, physical and educational standard’ of a given district.^106^ Ten years on, predelinquency had mobilised community anxieties in the mapping, monitoring and prognosis of behavioural risk, however, plans to evaluate ‘treatment’ had yet to be implemented.

Though the fate of Berkeley’s interwar crime prevention programme remains ambiguous, two of its core principles still loom large in modern American policing: prediction and the community. Influential models of ‘community policing’ have attempted to foster public trust in police ‘problem solving’ just as historians have charted the ‘public service’ functions of past law enforcement.^107^ The invocation of community, however, carries moral force which may aim to reintegrate the potential offender as a reformed member or exclude them as an outside threat. Later twentieth-century therapeutic models like ‘Communities That Care’, highlight one such dynamic of assimilation into or removal from ‘conventional’ (white, middle-class, American) society.^108^ Conversely, putting forward their famous ‘Broken Windows’ theory of policing in 1982, James Wilson and George Kelling asked ‘how can the police strengthen the informal social-control mechanisms of natural communities in order to minimise fear in public places?’^109^ Police responsibilities, thus formulated, extend beyond known offenders to the forceful control of the ‘disorderly’ and ‘disreputable, obstreperous or unpredictable people’ to maintain public standards against the threat of apathy, minor infractions, community breakdown and, ultimately, crime.^110^

Underlying notions of ‘natural community’ similarly inform the development and application of modern predictive policing software. Relying on the aggregation and algorithmic analysis of police reports and other sources of data, recent products such as PredPol, HunchLab and Crime Radar have been presented by proponents as ‘eminently technical and politically agnostic’.^111^ However, critical scholarship instead points to the role of these predictive policing technologies in reproducing unjust patterns of suspicion, surveillance and over-policing of racially marginalised groups. Once again, mapping crime and its apparent risk carries implications of where dangerous or protected space begins and ends, illustrated in the targeted application of predictive software in ‘problem neighbourhoods’, or ‘correction’ of algorithms to align with prior expectations of likely crime.^112^ R. Joshua Scannell thus describes HunchLab’s mapping of future crime and assignment of pre-emptive ‘missions’ based on the correlation of police reports with location, economic indices and even local fast food outlets. Police patrols and data collection reciprocally mark out risky (i.e. marginal, non-white) communities for intervention where ‘gastronomy and budget transform into criminality and risk, thereby mathematising and forecasting the “community” euphemism’.^113^ Notably, whereas ‘predelinquency’ suggested (qualified) therapeutic ambitions, the exclusionary logic of public safety has become paramount in recent prediction and profiling, used to justify the incarceration or ‘incapacitation’, of offenders who will apparently always be high-risk.^114^

Rebecca Lemov, in her call for a ‘history of precrime’, points to the need for alternatives to stories of ‘instrument-based determinism’ currently fixated on the pre-emptive or discriminatory potential of computerisation.^115^ The case of Berkeley, offering one such trajectory, shows how the community shaped suspicion. Vollmer, sharing a psychiatric notion of predelinquency with other practitioners, hoped that risks could be mapped and controlled, however, his programme differed markedly from later computerised prediction. The information networks that connected school, police, CPD and others in the Coordinating Council were personal and highly varied in their expectations or priorities of dealing with youth. Wider public participation in prediction, however, did not preclude the exercise of police and state power to excise or contain perceived foreign, dysfunctional or ‘defective’ threats. In turn, oversight of malleable juveniles expanded, along with anxiety to ensure their compliance with social, moral and mental norms so that they might participate in the future community. Suspicion towards broken windows, portions of a heatmap or other algorithmically identified risks are also ways of reproducing and reinforcing such boundaries. Communities appear, alternately, as sites of risk, fields of information and intelligence gathering, and models for corrective assimilation, all of which continue to shape the contours of suspicion and sympathy for delinquent or potentially delinquent youth.

## Footnotes

1 August Vollmer, ‘Predelinquency’, *Journal of Criminal Law and Criminology*, 1923, 14, 279–83, 281.

2 David Armstrong, ‘The Rise of Surveillance Medicine’, *Sociology of Health and Illness*, 1995, 17, 393–404, 395.

3
*Ibid*., 403.

4 Richard V. Ericson and Kevin D. Haggerty, *Policing the Risk Society* (Toronto: University of Toronto Press, 1997), 8.

5 Nicole Hahn Rafter, *Creating Born Criminals* (Urbana: University of Illinois Press, 1997), 9; See also Cesare Lombroso, *Criminal Man*, Mary Gibson and Nicole Hahn Rafter (trans), (Durham: Duke University Press, 2006 [1876–1897]); Gibson, *Born to Crime: Cesare Lombroso and the Origins of Biological Criminology* (Westport: Praeger, 2002); Michael Rembis, *Defining Deviance: Sex, Science and Delinquent Girls* (Urbana: University of Illinois Press, 2013); Miroslava Chavez-Garcia, *States of Delinquency: Race and Science in the Making of California’s Juvenile Justice System* (Berkeley: University of California Press, 2012).

6 Sol Cohen, ‘The Mental Hygiene Movement, The Development of Personality and the School: The Medicalization of American Education’, *History of Education Quarterly*, 1983, 23, 123–49; Gerald Grob, *Mental Illness and American Society, 1875–1940* (Princeton: Princeton University Press, 1983), 144–78; Kathleen Jones, *Taming the Troublesome Child: American Families, Child Guidance and the Limits of Psychiatric Authority* (Cambridge: Harvard University Press, 1999); Margo Horn, *Before It’s Too Late: The Child Guidance Movement in the United States, 1922–1945* (Philadelphia: Temple University Press, 1989); Matthew Smith et al., ‘Introduction’, in Despo Kritsotaki, Vicky Long and Matthew Smith, eds, *Preventing Mental Illness: Past, Present and Future* (London: Palgrave Macmillan: 2019), 1–38, 13–17.

7 Horn, *Before It’s Too Late*,

8 Chavez-Garcia, *States of Delinquency*, 47–70.

9 Theo Di Castri, ‘The Settler Colonial Roots and Neoliberal Afterlife of Problem Behaviour Theory’, *Journal of the History of the Behavioural Sciences*, 2023, 59, 107–28.

10 William Healy, ‘The Prevention of Delinquency and Criminality, *Journal of Criminal Law and Criminology*, 1933, 24, 74–87, 80–1.

11 O.W. Wilson, ‘August Vollmer’, *The Journal of Criminal Law, Criminology, and Police Science*, 1953, 44, 91–103.

12 Willard Oliver, *August Vollmer: The Father of American Policing* (Durham: Carolina Academic Press, 2017).

13 Lawrence Sherman, ‘Foreword’ in *Ibid*., xi.

14 Alexandra Minna Stern, *Eugenic Nation: Faults and Frontiers of Better Breeding in Modern America* (Oakland, University of California Press, 2015), 139–42; Julian Go, ‘The Imperial Origins of American Policing: Militarization and Imperial Feedback in the Early 20^th^ Century’, *American Journal of Sociology*, 2020, 125, 1193–254.

15 Michel Foucault, *Discipline and Punish: The Birth of the Prison*, Allen Lane (trans) (London: Penguin, 2020 [1975]), 177.

16 Kevin D. Haggerty and Richard V. Ericson, ‘The Surveillant Assemblage’, *British Journal of Sociology*, 2000, 51, 605–22; Bruno Latour, *Science in Action: How to Follow Scientists and Engineers Through Society* (Cambridge: Harvard University Press, 1987).

17 Bruno Latour, *Pandora’s Hope: Essays on the Reality of Science Studies* (Cambridge: Harvard University Press, 1999), 311.

18 Geoffrey Bowker and Susan Leigh Star, *Sorting Things Out: Classification and its Consequence* (Cambridge: MIT Press, 1999), 16.

19 Albert Schneider and August Vollmer, ‘The School for Police as Planned at Berkeley’, *Journal of the American Institute of Criminal Law and Criminology*, 1917, 7, 877–98, 878.

20 Julia Liss and Steven Schlossman, ‘The Contours of Crime Control in August Vollmer’s Berkeley’, *Research in Law, Deviance and Social Control*, 1984, 6, 79–107, 81.

21 A. Vollmer to C. Ruess, 7 December 1910, Box 18 folder 10, Berkeley Police Dept. Records 1909–32, Bancroft Library, University of California Berkeley (henceforth BPDR); Vollmer to Ruess, 20 November 1912, Box 19 folder 9, BPDR; Alfred Parker, *The Berkeley Police Story* (Springfield: Charles C. Thomas, 1972), 54.

22 ‘Criminology Courses, University of California Summer Session 1920’, 16, Carton 3 Folder 18, Jau Don Ball Papers, 1917–34, Bancroft Library, University of California Berkeley (henceforth JDBP).

23 Jau Don Ball and A.M. Kidd, ‘The Relation of Law and Medicine in Mental Diseases’, Pt. 1, *California Law Review*, 1920, 9, 1–26; Ball and Kidd, ‘The Relation of Law and Medicine in Mental Diseases’ Pt. 2, *California Law Review*, 1921, 9, 100–16; Ball and Kidd, ‘The Relation of Law and Medicine in Mental Diseases’ Pt. 3, *California Law Review*, 1921, 9, 276–305.

24 Vollmer to A.C. Matthews, 9 February 1920, Box 29 Folder 2, BPDR; Vollmer to R. R. Veale, 7 March 1919, Box 28 Folder 3, BPDR.

25 Vollmer to Ball, 22 March 1919, Box 28 Folder 3, BPDR.

26 Vollmer to A. Warren Stearns, 15 August 1919, Box 28 Folder 7, BPDR.

27 Vollmer, ‘Predelinquency’, 280.

28
*Ibid*., 281–2.

29
*Ibid*., 282.

30
*Ibid*., 283.

31 Margo Horn, ‘The Moral Message of Child Guidance 1925–1945’, *Journal of Social History*, 1984, 18, 25–36, 25; For British applications of the ‘American model’ of child guidance see John Stewart, ‘The Dangerous Age of Childhood: Child Guidance and the “Normal” Child in Great Britain, 1920–1950’, *Paedogogica Historica*, 2011, 47, 785–803.

32 Jones, *Taming the Troublesome Child*, 97, 91–147.

33 Horn, *Before It’s Too Late,* 42.

34 William A. White, ‘Underlying Concepts in Mental Hygiene’, *Mental Hygiene*, 1917, 1, 7–15.

35 Jau Don Ball, ‘Criminology (113 A) Lecture I’, 3, Carton 3 Folder 1, JDBP; Ball to Vollmer, 20 March 1919, Box 2 Folder 16, BPDR; Ball to Vollmer, 20 January 1921, Box 2 Folder 16, BPDR.

36 Vollmer, ‘Predelinquency’, 281.

37 Vollmer to C. Owings, 6 October 1924, Box 33 Folder 7, BPDR.

38 Vollmer to John Edy, 12 February 1925, Box 34 Folder 2, BPDR.

39 See Stern, *Eugenic Nation*, 1–27; Ian Dowbiggin, ‘A Rational Coalition: Euthanasia, Eugenics and Birth Control in America, 1940–1970’, *The Journal of Policy History*, 2002, 13, 223–60.

40 See William Healy, *The Individual Delinquent: A Text-Book of Diagnosis and Prognosis for All Concerned in Understanding Offenders* (Boston: Little, Brown & Co., 1915); Jones, *Taming the Troublesome Child*, 38–61.

41 For sociological and social psychiatric approaches see Matthew Smith, *The First Resort: The History of Social Psychiatry in the United States* (New York: Columbia University Press, 2023), 63–105.

42 See Matthew Smith, ‘Snips and Snails and Puppy Dog Tails: Boys and Behaviour in the USA’, *Canadian Bulletin of Medical History*, 2019, 36, 51–79; Nina Mackert, ‘Danger and Progress: White Middle-Class Juvenile Delinquency and Motherly Anxiety in the Post-War US’, in Heather Ellis (ed.), *Juvenile Delinquency and the Limits of Western Influence, 1850–2000* (Basingstoke: Palgrave Macmillan, Basingstoke), 199–223.

43 Parker, *The Berkeley Police Story*, 85.

44 Vollmer to Ball, 26 April 1919, Box 28 Folder 4, BPDR.

45 Vollmer to Queen Layer, 27 August 1919, Box 28 Folder 7, BPDR.

46 Vollmer to J. F. Fidler, 19 November 1920, Box 29 Folder 7, BPDR.

47 Vollmer to M. Brady, 11 September 1920, Box 29 Folder 6, BPDR.

48 Vollmer to L.C. Weymouth, 20 July 1921, Box 30 Folder 7, BPDR.

49 Jones, *Taming the Troublesome Child*, 3.

50 Vollmer to Elizabeth Anderson, 22 December 1924, Box 33 Folder 9, BPDR; For the influence of psychoanalytic theories of parent–child relationships on child guidance see Horn, *Before It’s Too Late*; Jones, *Taming the Troublesome Child*.

51 Elise H. Martens and Helen Russ, *Adjustment of Behavior Problems of School Children: A Description and Evaluation of the Clinical Program in Berkeley, Calif* (Washington DC: United States Department of the Interior, Office of Education, 1932), 7.

52 ‘Coordinating the Agencies of the City of Berkeley in Behalf of Youth’ (1927), 3, Vol. 1 Virgil E. Dickson Papers, 1920–45, Bancroft Library, University of California Berkeley (Henceforth VEDP).

53 Herman Adler to Vollmer, 20 November 1925, Box 1 Folder 1, BPDR.

54 Vollmer to Adler, 24 November 1925, Box 34 Folder 10, BPDR.

55 Liss and Schlossman, ‘The Contours of Crime Control’, 79–107.

56 Adler to Vollmer, 20 November 1925, Box 1 Folder 1, BPDR.

57 Vollmer to Agnes Stillson, 5 January 1920, Box 29 Folder 2, BPDR.

58 Vollmer to Herb Spencer, 11 May 1925, Box 35 Folder 5, BPDR; Vollmer to Marion Clark, 11 March 1927, BPDR.

59 Martens and Russ, *Adjustment of Behaviour Problems of School Children*, 5.

60 Elizabeth Lossing, ‘The Crime Prevention Work of the Berkeley Police Department’, in Eleanor and Sheldon Glueck, eds, *Preventing Crime: A Symposium* (New York: McGraw-Hill Book Company, 1936), 237–63, 237.

61 Go, ‘The Imperial Origins of American Policing’, 1217.

62 Vollmer to D. J. O’Brien, 8 March 1927, Box 36 Folder 6, BPDR.

63 Martens and Russ, ‘Adjustment of Behavior Problems of School Children’, 8; See also, James W. Trent Jr., *Inventing the Feeble Mind: A History of Mental Retardation in the United States* (Berkeley: University of California Press, 1994).

64 Martens and Russ, ‘Adjustment of Behavior Problems of School Children’, 14.

65 ‘Report Given Before the Berkeley Board of Education’ (1921), 2, Vol. I, VEDP.

66 ‘Behaviour Research Program in the Berkeley Public Schools’ (1930), 3–6, Vol. I, VEDP.

67
*Ibid*., 1.

68 ‘Counselling in the Secondary Schools’ (1928), 1, Vol. 1, VEDP.

69 ‘Behaviour Research Program in the Berkeley Public Schools’ (1930), 3–6, Vol. I, VEDP. 8–10.

70 ‘Counselling in the Secondary Schools’ (1928), 5, Vol. I, VEDP.

71
*Ibid*., 11–13.

72 Martens and Russ, ‘Adjustment of Behavior Problems of School Children’, 36–40.

73
*Ibid*., 40.

74 See Theodore Porter, *Trust in Numbers: The Pursuit of Objectivity in Science and Public Life* (Princeton: Princeton University Press, 1995).

75 ‘Behavior Difficulties that Baffle Teachers’ (1931), 3, Vol. I, VEDP.

76 ‘Overt Problem Behavior’ (1931), 4, Vol. I, VEDP.

77
*Ibid*., 1.

78
*Ibid*., 8.

79
*Ibid*., 6–7.

80 ‘Mind Ways First Radio Talk’ (1932), Vol. II, VEDP.

81 Jones, *Taming the Troublesome Child*, 146.

82 ‘Why is John a Loyal, Patriotic Citizen?’ (1934), Vol. II, VEDP.

83 ‘Is One Born to be a Criminal?’ (1936), Vol III, VEDP.

84 Vollmer to Elizabeth Anderson, 22 December 1924, Box 33 Folder 9, BPDR.

85 Vollmer to Anderson, 8 May 1925, Box 34 Folder 5, BPDR.

86 Lossing, ‘The Crime Prevention Work of the Berkeley Police Department’, 256.

87
*Ibid*., 253.

88
*Ibid*., 254.

89
*Ibid*., 253–5.

90 Vollmer to Owings, 6 October 1924, Box 33 Folder 7, BPDR.

91 David Wolcott, *Cops and Kids: Policing Juvenile Delinquency in Urban America, 1890–1940* (Columbus: Ohio State University Press, 2005).

92 Lossing, ‘The Crime Prevention Work of the Berkeley Police Department’, 258–9.

93
*Ibid*., 250; 253.

94
*Ibid*., 256–7.

95
*Ibid*., 250.

96 Vollmer to M. Newmark, 8 April 1925, Box 34 Folder 4, BPDR.

97 Lossing, ‘The Crime Prevention Work of the Berkeley Police Department’, 250.

98 ‘BPD’ Case Notes, 28–29, Carton 1 Folder 10, JDBP.

99
*Ibid*., 11.

100
*Ibid*., 13.

101
*Ibid*., 12.

102 William Shepard to Vollmer, 6 November 1925, Box 3 Folder 9, BPDR.

103 Lossing, ‘The Crime Prevention Work of the Berkeley Police Department’, 251.

104
*Ibid*., 254.

105
*Ibid*., 262.

106 Vollmer to Anderson, 8 May 1925, Box 34 Folder 5, BPDR.

107 Michael Reisig, ‘Community and Problem-Oriented Policing’, *Crime and Justice*, 2010, 39, 1–53; Eric Monkonnen, ‘History of Urban Police’, *Crime and Justice*, 1992, 15, 547–80.

108 Di Castri, ‘The Settler-Colonial Roots and Neoliberal Afterlife of Problem Behaviour Theory’, 107–28.

109 James Wilson and George Kelling, ‘Broken Windows’, *The Atlantic*, March 1982, 29–38, 35.

110
*Ibid*., 30.

111 Daniel Edler Duarte, ‘The Making of Crime Predictions: Sociotechnical Assemblages and the Controversies of Governing Future Crime’, *Surveillance and Society*, 2021, 19, 202.

112 See *Ibid*., 199–215; Andrea Miller, ‘Shadows of War, Traces of Policing: The Weaponization of Space and the Sensible in Preemption’, in Ruha Benjamin, ed., *Captivating Technology: Race, Carceral Technoscience, and Liberatory Imagination in Everyday Life* (Durham: Duke University Press, 2019), 85–106.

113 R. Joshua Scannell, ‘This is Not *Minority Report: Predictive Policing and Population Racism*’, in Ruha Benjamin, ed., *Captivating Technology*, 117.

114 See Benjamin Harcourt, *Against Prediction: Profiling and Punishing in an Actuarial Age* (Chicago: University of Chicago Press, 2007), 145–71.

115 Rebecca Lemov, ‘An Episode in the History of PreCrime’, *Historical Studies in the Natural Sciences*, 2018, 48, 637–47.

## Notes

John Shepherd received his BA in History in 2017 and MPhil in the History of Science, Medicine and Technology in 2019, both from the University of Oxford, and in 2024 he completed his PhD in Durham University's Department of Philosophy. His thesis examines attempts to pre-empt future crime and juvenile delinquency in the twenthieth-century USA through methods of psychiatric prognosis and probabilistic prediction. His current research project focuses on the postwar development of computer crime mapping.

